# Lysosome-Dependent Activation of Human Dendritic Cells by the Vaccine Adjuvant QS-21

**DOI:** 10.3389/fimmu.2016.00663

**Published:** 2017-01-05

**Authors:** Iain Welsby, Sophie Detienne, Francisca N’Kuli, Séverine Thomas, Sandrine Wouters, Viviane Bechtold, Dominique De Wit, Romain Gineste, Thomas Reinheckel, Abdelatif Elouahabi, Pierre J. Courtoy, Arnaud M. Didierlaurent, Stanislas Goriely

**Affiliations:** ^1^Institute for Medical Immunology, Université Libre de Bruxelles (ULB), Gosselies, Belgium; ^2^Cell Biology Unit, de Duve Institute, Université Catholique de Louvain, Brussels, Belgium; ^3^GSK Vaccines, Rixensart, Belgium; ^4^Medical Faculty, Institute for Molecular Medicine and Cell Research, Albert Ludwigs University, Freiburg, Germany

**Keywords:** vaccine, adjuvant, saponins, lysosome, cathepsins, dendritic cells, cytokine

## Abstract

The adjuvant properties of the saponin QS-21 have been known for decades. It is a component of the Adjuvant System AS01 that is used in several vaccine candidates. QS-21 strongly potentiates both cellular and humoral immune responses to purified antigens, yet how it activates immune cells is largely unknown. Here, we report that QS-21 directly activated human monocyte-derived dendritic cells (moDCs) and promoted a pro-inflammatory transcriptional program. Cholesterol-dependent QS-21 endocytosis followed by lysosomal destabilization and Syk kinase activation were prerequisites for this response. Cathepsin B, a lysosomal cysteine protease, was essential for moDC activation *in vitro* and contributed to the adjuvant effects of QS-21 *in vivo*. Collectively, these findings provide new insights into the pathways involved in the direct activation of antigen-presenting cells by a clinically relevant QS-21 formulation.

## Introduction

Purified recombinant antigens generally elicit weak immune responses in vaccines unless they are co-formulated with adjuvants. Adjuvants stimulate the innate immune response, which in turn shapes cell-mediated and humoral responses that eventually confer protection against pathogens. Saponins are a class of molecules that possess adjuvant activity. QuilA, a mixture of saponins extracted from the bark of the *Quillaja saponaria* tree, is a potent adjuvant that stimulates cell-mediated and humoral responses (1). A water-soluble triterpene glycoside purified from this mixture, QS-21, possesses strong adjuvant activity and limited toxicity when compared to the other fractions (2). In animal models, QS-21 is a potent inducer of humoral, Th1 CD4 and cytotoxic CD8 T cell responses (3–5). CD8 T cell induction is most likely due to antigen cross-presentation, as has been shown for other saponins (6, 7). QS-21 possesses hemolytic activity and thereby intrinsic toxicity, due to saponin interaction with membrane cholesterol that leads to pore formation (2, 8). Abrogation of the lytic activity of QS-21 is achieved by co-formulation with cholesterol (9), a process used in the Adjuvant System AS01 (GSK) that results in reduced toxicity and adverse reactions (10).

Activation of innate cells by adjuvants can shape the subsequent adaptive responses. For example, alum stimulates innate cells to produce cytokines such as IL-4 and IL-6 that could explain the polarization toward Th2 responses in mice (11). On the other hand, toll-like receptor (TLR) ligands, such as CpG oligodeoxynucleotides, induce the production of IL-12 by innate cells thereby favoring Th1 responses (12). The activation of different pattern recognition receptors can lead to the production of various cytokines and thus alter the adaptive response. It is therefore crucial to understand the early pathways activated by adjuvants as this step can have profound effects on the quality of the adaptive response. The mechanisms by which saponins activate antigen-presenting cells are still poorly understood. Both QuilA and QS-21 activate the NLRP3 inflammasome *in vitro* (13, 14). Furthermore, ISCOMATRIX, an adjuvant containing different QuilA fractions also activates the inflammasome *in vitro* and *in vivo* (15). However, the antigen-specific responses elicited by QS-21-containing vaccines were found to be largely independent of the NLRP3 inflammasome pathway, suggesting that other mechanisms must be involved in its adjuvanticity (14, 15).

Here, we show that QS-21 directly activated human monocyte-derived dendritic cells (moDC) and identified specific signaling pathways that lead to acute transcriptional activation. We found that QS-21 was endocytosed in a cholesterol-dependent manner and accumulated in lysosomes. We further demonstrate that lysosomal destabilization and cathepsin B activity were central for the response of moDCs to QS-21. We also show that absence of cathepsin B decreased antigen-specific CD4 and CD8 T cell responses in a murine vaccination model.

## Materials and Methods

### Cells and Reagents

THP-1 (ATCC: Tib202) cells were cultured in medium (RPMI containing 10% FCS, l-glutamine, pyruvate, non-essential amino acids, 2-mercaptoethanol, and penicillin/streptomycin/gentamycin) and differentiated into macrophages by 200 nM phorbol myristate acetate (PMA) for 3 days, then further cultured without PMA for 4–5 days. QS-21 (*Quillaja saponaria* Molina, fraction 21; licensed by GSK from Antigenics LLC, a wholly owned subsidiary of Agenus Inc., a Delaware corporation) and hepatitis B surface antigen (HBsAg) were provided by GSK Vaccines. For the experiments, QS-21, BODIPY QS-21, and ^14^C-QS-21 were formulated in DOPC liposomes containing cholesterol at 100 µg/ml. The term “QS-21” used in the manuscript refers to QS-21 formulated in liposomes. The following reagents were used at the indicated concentrations or following manufacturer’s indications: Z-VAD-FMK (10 µg/ml—Invivogen), methyl-β-cyclodextrin (1/2.5/5/10 μM—Sigma-Aldrich), Bay 61 3606 (10 µM—Sigma-Aldrich), Bafilomycin A1 (BafA1) (250 nM—Santa Cruz Biotechnology), acridine orange (AO) (1 µg/ml—Sigma-Aldrich), CA-074 Me (10 µM—PeptaNova), Z-FF-FMK (20 µM—Santa Cruz Biotechnology), Z-FA-FMK (20 µM—Santa Cruz Biotechnology), pepstatin A (10 µM—Santa Cruz Biotechnology), and zymosan (50 µg/ml—Invivogen). For confocal studies, QS-21 was chemically linked to the green fluorescent dye BODIPY (4,4-difluoro-4-bora-3a,4a-diaza-s-indacene). To label QS-21, an ester derivative of QS-21 (utilizing the glucuronic acid group) was prepared by treating QS-21 (in dimethylformamide) with sulfo-*N*-hydroxysuccinimide and dicyclohexylcarbodiimide, as described previously (16). The QS-21-sulfo-NHS ester derivative was purified by precipitation with ethyl acetate, then dried and suspended in 0.1 M NaHPO4 buffer (pH7), and reacted with an excess of BODIPY FL-EDA (which bears an amine group; D2390, Life Technologies). The reaction product, BODIPY-QS-21, was purified by semi-preparative HPLC and isolated as a powder. For endocytosis assays, the ^14^C-QS-21 was synthesized by GE Healthcare. The activity of these analogs was confirmed by a hemolysis assay.

### moDC Generation

Buffy coats from healthy donors who consented to their use for scientific research were obtained from local blood donations. The study received approval from the Hôpital Erasme Ethics Committee (Route de Lennik 808, B-1070 Brussels). Peripheral blood mononuclear cells (PBMCs) were purified by density gradient centrifugation (Lymphoprep—Stemcell Technologies). CD14^+^ monocytes were purified from PBMCs by positive magnetic separation with anti-CD14 microbeads (Automacs—Miltenyi Biotec) and cultured in 75-cm^2^ flasks with 20 ml of medium (RPMI containing 10% FCS, penicillin/streptomycin, non-essential amino acids, and glutamine) supplemented with recombinant human GM-CSF (800 U/ml—Gentaur) and IL-4 (200 U/ml—Wittycell). After 3 days, 16,000 U GM-CSF and 4,000 U IL-4 were added to the medium. At day 6–7, non-adherent cells corresponding to DCs (>90% CD14-negative HLA-DR-positive CD11c-positive) were recovered, washed, and used for subsequent experiments.

### Mice

Six-week-old C57BL/6 mice were purchased from Harlan Horst. Cathepsin B-deficient mice (*Ctsb*^−/−^) were previously described (17). These mice were housed and bred under specific pathogen-free conditions. Animal studies were approved by the institutional animal care and local committee for animal welfare.

### Mice Immunization

Six-week-old female mice received injections containing 4 µg of HbsAg, 1 µg of ovalbumin (OVA) and 1 µg of QS-21 in the gastrocnemius muscles of both hind limbs in a volume of 20 µl per muscle. OVA was obtained from Calbiochem and confirmed to be endotoxin-free.

### Endocytosis Assay

PMA-differentiated THP-1 cells were incubated at 37°C for 1 or 4 h with 1 or 10 µg/ml ^14^C-QS-21, as appropriate, surface-stripped by limited trypsin digestion, pelleted, washed, and lysed. ^14^C radioactivity (Tri-Carb 2800TR, Perkin Elmer) was considered as intracellular QS-21 and normalized to cell protein. ATP was depleted by preincubation with 10 mM azide and 50 mM deoxyglucose in RPMI medium without FCS for 20 min. Cholesterol was depleted by preincubation with 2.5 mM methyl-β-cyclodextrin for 4 h, and then, cells were extensively washed prior tracer uptake. This resulted into marginal (5–10%) cell cholesterol loss. Endocytosis of ^125^I-transferrin or -cholera toxin B subunit was performed for 15 min, then cells were stripped with trypsin and ^125^I counts in lysates were measured with a gamma counter, as described in Ref. (18) (except that pronase was replaced by 1% trypsin). Uptake was saturable with ^14^C-QS-21 concentration around 10 µg/ml. Accumulation was linear with time for at least 6 h indicating lack of degradation and effective retention, which was further confirmed by the negligible loss after a 2-h chase. ^14^C-QS-21 integrity was confirmed by TLC/phosphorimager.

### Subcellular Fractionation

After ^14^C-QS-21 accumulation for 4 h and chase overnight, cells were surface stripped with trypsin or not, homogenized, and submitted to differential sedimentation to yield postnuclear particles as described in Ref. (19). These were mixed with 15% Percoll to form a self-generating gradient. Ten fractions of 1 ml were collected from the bottom and analyzed for radioactivity (stripped: recovery, 80% of homogenate; non-stripped, 50%), beta-hexosaminidase activity (stripped or not; >80%), and Western blotting for Na^+^/K^+^-ATPase (non-stripped cells).

### Microscopy

For BODIPY-QS-21 imaging, 10^5^ moDCs were seeded in each compartment of compartmented 35-mm sterile culture dishes with glass bottom (Cellview—Greiner). Cells were incubated for 1 h with 10 µg/ml BODIPY-QS-21 incorporated in liposomes in complete medium, transferred into RPMI without phenol red, and analyzed by confocal vital imaging (Zeiss LSM710 confocal). For fluorescent tracer uptake, cells were seeded as above, incubated with 1 mg/ml lucifer yellow, 50 µg/ml 10 kDa dextran-Alexa647, and 50 µg/ml 40 kDa dextran-Texas Red for 16 h, washed, stimulated with QS-21-containing liposomes for 4 h, and analyzed by confocal vital imaging. For NF-κB p65 nuclear translocation, p65 was detected following a classical immunofluorescence protocol described previously (20) using goat anti-p65 (Santa Cruz Biotechnology sc-372x—1/1,500 in PBS added with 0.1% Tween 20) and donkey anti-goat AlexaFluor594 (Invitrogen A11058—1/1,000) antibodies, and then visualized on a Zeiss LSM710 confocal microscope.

### Flow Cytometry

A total of 10^6^ moDCs were activated overnight with the indicated stimulants. They were then washed in FACS buffer, resuspended in 50 µl, and FC receptors were blocked with human FcR Blocking Reagent (Miltenyi). Cells were then incubated with CD86-APC and HLA-DR-PE-Cy7 (eBioscience, 1/100). For innate cell recruitment to the draining lymph node, DLNs were treated by mechanical dissociation in 3 ml DMEM containing DNase I (100 mg/ml; Roche) and Liberase (0.26 U/ml; Roche) for 30 min. The cells were passed through a 100 mM cell strainer, resuspended in FACS buffer containing FC-blocking reagent (1/50; 2.4G2; BD Biosciences) and stained with: anti-Ly6C-APC, the PE-conjugated lineage identifiers (anti-SiglecF, anti-CD90.2, anti-CD19, and anti-NK1.1), anti-Ly6G-PerCPCy5, anti-Ly6C-APC, anti-MHC class II (MHCII) (I-A/I-E)-FITC (eBioscience), anti-CD11b-V450, and anti-CD11c-PECy7. All Abs except the anti MHCII-FITC were obtained from BD Biosciences. To assess antigen (Ag)-specific T cell response, splenocytes from immunized mice were stimulated *in vitro* in a 96-well microplate with HBs peptide pools (GSK) at 1 µg/ml in the presence of anti-CD49d and anti-CD28 Abs, both 1 µg/ml (BD Biosciences). After 2 h, cytokine secretion was inhibited with 1 µg/ml brefeldin A (BD Biosciences), and cells were cultured overnight at 37°C. The cell were resuspended in FACS buffer containing Fc-blocking reagent (1/50; 2.4G2; BD Biosciences) and extracellular receptors were immunolabeled (Anti-CD4 pacific blue, clone RM 4-5 and anti-CD8-PerCP, clone 53-6.7) before permeabilization with Cytofix–Cytoperm (BD Biosciences). Cells were immunolabeled with anti-IFN-γ-APC (clone XMG1.2), anti-IL-2-FITC (clone JES6-5H4), and anti-TNF-PE (clone MP6-XT22). All antibodies were from BD Biosciences.

### Microarray Analysis

A total of 10^6^ moDCs from four donors were stimulated with QS-21 for 4 h in complete medium. Cells were washed with PBS, scraped, and lysed in 1 ml Tripure reagent (Roche Applied Science). RNA was extracted with chloroform and purified with the RNeasy Minikit (Qiagen) following manufacturer’s instructions, with DNase treatment of the column to remove possible genomic DNA contamination. RNA was concentrated by ethanol precipitation followed by resuspension in RNase-free water. RNA was quantified with a NanoDrop spectrophotometer. Biotinylated aRNA was generated from 100 ng total RNA using a Genechip 3′IVT express kit (Affymetrix, Santa Clara, CA, USA). Samples (10 µg biotinylated and fragmented aRNA) were hybridized on a HG-U133A 2.0 Expression chip (Affymetrix) for 16 h. Arrays were washed according to manufacturer’s recommendation and scanned using the Genechip scanner 3000 Affymetrix. Fold changes [QS-21 or 3-*O*-desacyl-4′-monophosphoryl lipid A (MPL; GSK Vaccines) vs. medium] were calculated for each donor and a heatmap of top differentially expressed genes (FC > 5 or <0.5) was generated with MeV software. A volcano plot was generated with the average fold change and *p*-values for each gene using GraphPad Prism software. A scatter plot of fold changes vs. medium of QS-21 (*x* axis) and MPL (*y* axis) was generated using the GraphPad Prism software.

### Quantitative Real-time PCR

mRNA content of 2 × 10^5^ moDCs was isolated with the MagNA Pure LC mRNA isolation kit (Roche) on the MagNA pure instrument (Roche Applied Science) following manufacturer’s indications. TNF, IL-6, IL-8, and IL-1β mRNAs were quantified by real-time PCR using TaqMan RNA Amplification Kit (Roche) or LightCycler Multiplex RNA Virus Master (Roche) on a LightCycler 480 instrument (Roche Applied Science). Primers and probes were synthesized by Eurogentec (Actb: F: ggatgcagaaggagatcactg, R: cgatccacacggagtacttg, probe: ccctggcacccagcacaatg; Ctsb: F: tcccaccatcaaagagatca, R: atgcagatccggtcagagat, probe: tgtggctcctgctgggcctt; Syk: F: cagggaatatgtgaagcagaca, R: tccagctgaggcttctgact, probe tcaggctctggagcaggcca; IL1b: F: acagatgaagtgctccttcca, R: gtcggagattcgtagctggat, probe: ctctgccctctggatggcgg; IL6: F: gacagccactcacctcttca, R: agtgcctctttgctgctttc, probe: cctcgacggcatctcagccc; CXCL8/IL8: F: gccttcctgatttctgcagc, R: actgacatctaagttctttagcactcc, probe: tggcaaaactgcaccttcacacag, TNF: F: cccagggacctctctctaatc, R: atgggctacaggcttgtcact, probe: tggcccaggcagtcagatcatc). mRNA levels were normalized to β-actin mRNA expression.

### SDS-PAGE and Western Blotting

A total of 10^6^ moDCs were rinsed with PBS and lysed in 50 µl RIPA buffer (PBS with 1% Igepal CA-630, 0.5% Na deoxycholate and 0.1% SDS) containing protease (cOmplete Mini Protease Inhibitor Cocktail Tablet, Roche) and phosphatase inhibitors (PhosSTOP, Roche). Lysed cells were incubated on ice for 20 min, cleared by centrifugation at 12,000 *g* for 20 min at 4°C and stored at −80°C. Protein concentration was measured with the Micro BCA Protein Assay kit (Pierce) and 20 µg of protein was loaded onto a 12% Bis-Tris polyacrylamide gel. Gels were run in NuPAGE MOPS SDS Running Buffer (Invitrogen) at 150 V for 1 h. Proteins were transferred onto a PVDF membrane (Amersham) for 1 h at 100 V, blocked in TBS-Tween containing 5% BSA, and blotted with rabbit anti-phospho-Zap-70 (Tyr319)/Syk (Tyr352) (Cell Signaling #2701 1/1,000), rabbit anti-phospho-Syk (Tyr323) (Cell Signaling #2715 1/1,000), rabbit anti-Syk (Santa Cruz Biotechnology #sc-1077—1/500) and mouse anti-actin (Sigma-Aldrich clone AC-74—1/2,000) antibodies, followed by detection with donkey anti-rabbit/mouse IgG-HRP (GE Healthcare—1/5,000).

### ELISA

For cytokine measurements, 2 × 10^5^ moDCs were seeded in 96-well plates and allowed to rest for 2 h. Cells were treated with the appropriate inhibitors and stimulated. The supernatants were collected after 24 h and ELISAs (hIL-6, hIL-8, hTNF) were carried out following manufacturer’s instructions (R&D Systems). For Ag-specific antibody measurements, the HBs protein was used as the coating antigen and goat anti-mouse IgG (GAM—SouthernBiotech or Jackson ImmunoResearch) was used as the coating antibody for the standard curve. Plates were blocked at room temperature for 1 h (PBS–1% BSA) and serial dilutions of serum samples and the IgG standard (SouthernBiotech) were added and incubated for 2 h at room temperature, followed by addition of biotin-conjugated anti-mouse IgG (Jackson Research or SouthernBiotech) and streptavidin-HRP. TMB was used as a substrate and plates were read at 450 nm on a microplate reader.

### Virus Production and Transduction

pGIPZ vector-based short hairpin RNA (shRNA) targeting Syk (three constructs), cathepsin B (five constructs), and GAPDH as a control were purchased from Dharmacon. The following constructs exhibiting the highest knockdown capacity (verified by qPCR) for each target were selected (Syk: TCTATGATGTTCTTATCCT, cathepsin B: AACTTGACAGGGTGAAGCT). These vectors were transiently transfected into the human embryonic kidney HEK293T cells in combination with the packaging vector psPAX2 (Addgene plasmid 12259) and the VSV-G encoding plasmid pMD2.G as previously described (21). The supernatant was harvested 48 h after transfection and viruses were concentrated by ultracentrifugation and resuspension in Opti-MEM medium (Thermo Fisher). Viral titers were determined by transduction of 293 T cells with serial dilutions of the concentrated virus stock followed by flow cytometric analysis of GFP expression. SIV virion-like particles (SIV-VLPs) were produced by cotransfecting HEK293T cells with pMD2.G and pSIV3+ (22). moDCs were transduced as previously described (22, 23). Briefly, CD14^+^ monocytes were purified as described above, and seeded in 6-well plates in complete medium containing 800 U/ml GM-CSF, 200 U/ml IL-4, 1 µg/ml polybrene, and 100 µl of supernatant containing SIV-VLPs. After 1 h, lentiviral particles were added at a MOI of 1 and the plates were centrifuged for 1 h at 900 *g* at room temperature. On day 3, 1 ml of medium containing 2,400 U GM-CSF and 600 U IL-4 was added to each well. The cells were recovered on day 6, and the transduction efficiency (GFP expression) was measured by flow cytometry (typically, between 80 and 90% of cells were transduced).

### Statistical Analysis

Statistical analyses were performed using the GraphPad Prism software. Statistical significance for moDC data was determined either with a Wilcoxon matched-pairs signed-rank test or two-way ANOVA followed by Tukey’s multiple comparisons test. Statistical significance for murine *in vivo* data was assessed using a Mann–Whitney test. *p* ≥ 0.05: not significant; *p* < 0.05 and ≥0.01: *; *p* < 0.01 and ≥0.001: **; *p* < 0.001 and ≥0.0001: ***; *p* < 0.0001: ****.

## Results

### QS-21 Promotes the Activation and Maturation of Human moDCs

**Figure 1:**
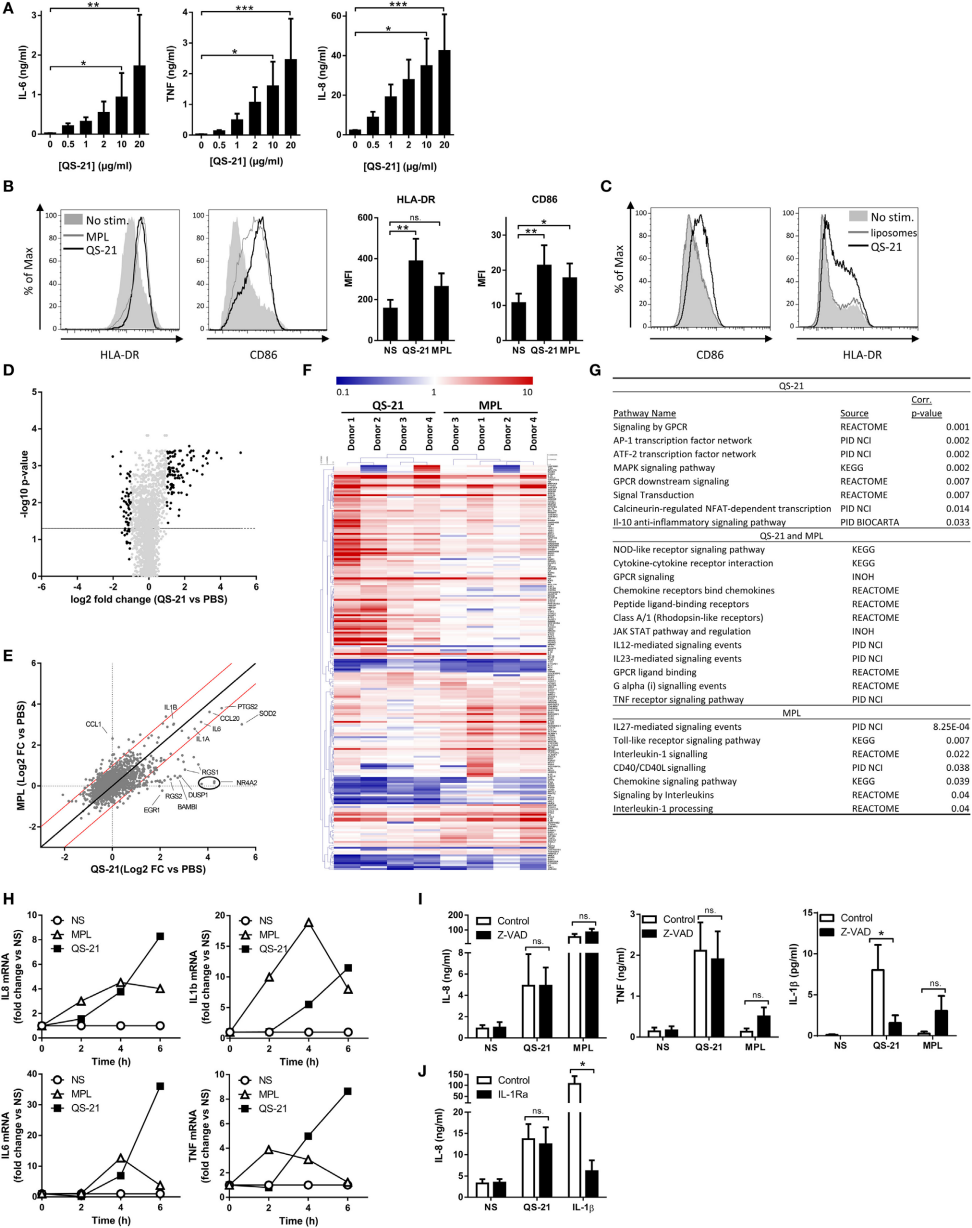
**QS-21 directly activates human dendritic cells**. **(A)** moDCs were stimulated with increasing concentrations of QS-21, and IL-6, TNF, and IL-8 were quantified in the culture supernatant after 24 h. Statistical significance was determined by a Friedman test followed by Dunn’s multiple comparisons. The means and SEM from four donors are shown. **(B)** moDCs were stimulated with QS-21 (10 µg/ml) or MPL (1 µg/ml) for 24 h, and surface marker expression was evaluated by flow cytometry. Representative histograms of HLA-DR and CD86 MFI and bar graphs representing the mean and SEM of cumulative data from eight donors are shown. **(C)** moDCs were stimulated with QS-21 (10 µg/ml) or liposomes for 24 h and CD86 and HLA-DR expression were evaluated by flow cytometry **(D–F)** moDCs from four donors were stimulated with QS-21 or MPL for 4 h and gene expression was analyzed by microarray. **(D)** Volcano plot of microarray data. Each point represents one gene plotted by log2 fold change (FC) vs. medium against −log10 of the *p*-value (average of four donors). The horizontal bar represents a *p*-value of 0.05. The light gray points represent genes with FC < 2 and FC > 0.5 and black points represent FC > 2 or FC < 0.5. **(E)** Scatter plot analysis of microarray data. Log transformed FC vs. medium of QS-21 is plotted against FC vs. medium of MPL (average of four donors). The dotted lines represent FC = 1, the solid black line represents FC_QS-21_ = FC_MPL_ and the red lines represent a FC_QS-21_/FC_MPL_ ratio of +2 or −2. **(F)** Hierarchical clustering and heatmap representation of differentially expressed genes (FC vs. medium >2 or <0.5) for the four donors. The color-coded scale representing fold change vs. medium (blue = downregulated vs. medium, red = upregulated vs. medium) is indicated at the top of the chart. **(G)** InnateDB analysis of pathway over-representation of significantly upregulated genes (*p*-value < 0.05 and fold change > 2) for cells stimulated with QS-21 (left) or MPL (right) using the hypergeometric algorithm and Benjamini–Hochberg correction for *p*-values. **(H)** moDCs were stimulated with QS-21 (10 µg/ml) or MPL (1 µg/ml) for the indicated durations, and IL-8, IL-1β, IL-6, and TNF transcript abundance was measured by qPCR. Data are from one donor representative of at least three independent experiments. **(I)** moDCs (*n* ≥ 5) were treated with Z-VAD-fmk (10 µM) and stimulated with QS-21 (10 µg/ml) or MPL (1 µg/ml), and IL-8, TNF, and IL-1β in the supernatant were measured by ELISA. **(J)** moDCs (*n* ≥ 5) were treated with recombinant IL-1 receptor antagonist (Anakinra—10 µg/ml) and stimulated with QS-21 (10 µg/ml) or IL-1β (10 ng/ml) and IL-8 was measured by ELISA. Statistical significance was determined by two-way ANOVA followed by Tukey’s multiple comparisons test.

QS-21 has been shown to activate the inflammasome in mouse macrophages and dendritic cells (14). In order to explore the signaling pathways involved in the immunostimulatory properties of saponins in human cells, monocyte-derived DCs (moDCs) were stimulated with increasing concentrations of QS-21 formulated into cholesterol-containing liposomes (hereafter referred to as “QS-21”), as in the clinical AS01 formulation. We observed dose-dependent induction of TNF, IL-6, and IL-8 and selected 10 µg/ml for following experiments (Figure 1A). The activation of moDCs by QS-21 or MPL, a TLR4 ligand, was analyzed by flow cytometry for the surface expression of the class II major histocompatibility complex (MHCII) molecule, HLA-DR, and the costimulatory receptor, CD86. Both proteins were upregulated similarly by either QS-21- or MPL-stimulated moDCs, indicating that QS-21 promotes moDCs activation (Figure 1B). Furthermore, this effect was dependent on the QS-21 component as the liposomes alone did not induce upregulation of these markers (Figure 1C). Next, moDCs from four donors were stimulated with either QS-21 or MPL for 4 h and global gene expression was analyzed by microarray. PBS-treated cells were used as the baseline to which both QS-21 and MPL treatments were compared. MPL was used as a control for its ability to uniquely activate TLR4 and to better discriminate QS-21-specific pathways.

In order to visualize changes in gene expression induced by QS-21, average microarray data were visualized on a “volcano plot” (Figure 1D). Overall, QS-21 induced significant upregulation of 102 probe sets and downregulation of 43 (Table S1 in Supplementary Material). Comparison of transcript expression profiles between QS-21 and MPL with a scatter plot identified co-regulated genes and genes specifically regulated by either QS-21 or MPL at this time point (Figure 1E). Genes upregulated by both stimulants included cytokine or chemokine genes such as *IL6, IL1A, IL1B, CXCL2, CXCL3*, or *CCL20*, demonstrating a common inflammatory core response as previously reported for other adjuvants (24). Top upregulated probe sets identified as specific to QS-21 (FC_QS-21_ > 2 and FC_MPL_ < 2) at this time point included *RGS1, RGS2, NR4A2, DUSP1*, and *EGR1*, which are primary response genes that are rapidly induced following cell activation with inflammatory stimuli (25–28). Clustering analysis of the top up- or downregulated genes for individual donors (FC vs. medium > 2 or FC vs. medium < 0.5) showed that individuals were clustered by stimulation (QS-21 vs. MPL) rather than by donor, indicating that the difference between MPL and QS-21 stimulation is greater than variations between different donors (Figure 1F). Pathway enrichment analysis with the InnateDB resource (29) identified pathways either shared by QS-21 and MPL, such as cytokine, Nod-like receptor (NLR), and G-protein-couple receptor signaling (Figure 1G), or specific to MPL such as TLR and IL-1 signaling, as expected (30). Pathways specific to QS-21 included AP-1 and ATF2 transcription factor networks, which mediate gene regulation in response to cytokines, stress signals, or infectious agents (31). Given that the QS-21-specific genes identified at this time point were known early response genes, we focused on the early expression kinetics of several upregulated cytokines shared by MPL and QS-21 by qPCR. Expression of these genes upon stimulation with QS-21 or MPL showed different kinetics, suggesting that slower or more complex pathways were triggered by QS-21 (Figure 1H). Finally, since saponins including QS-21 can activate the inflammasome and promote the release of IL-1β, we examined whether caspase activation or IL-1β signaling were required for cytokine production. Treatment of moDCs cells with the pan-caspase inhibitor Z-VAD-FMK had no effect on IL-8 or TNF production and, as expected, decreased IL-1β levels following QS-21 stimulation (Figure 1I). Furthermore, a recombinant IL-1 receptor antagonist had no effect on IL-8 production by cells stimulated with QS-21, but significantly decreased the response to IL-1β (Figure 1J). These results strongly suggest that cytokine production by QS-21-stimulated moDCs does not depend on inflammasome activation.

### Membrane Cholesterol Is Required for the Endocytosis of QS-21 and Activation of Immune Cells

**Figure 2:**
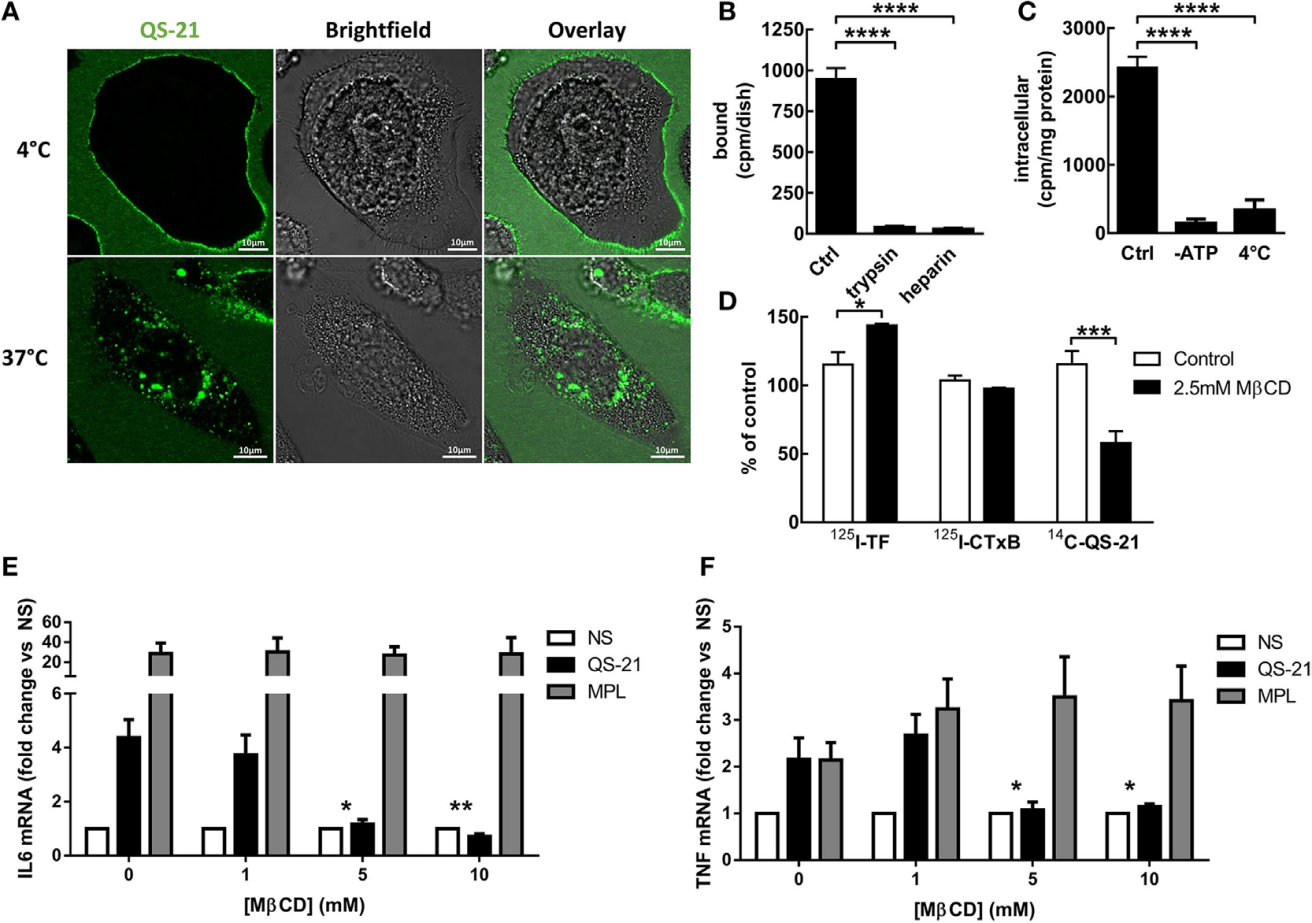
**QS-21 is internalized by cholesterol-dependent endocytosis**. **(A)** *Imaging*: THP-1 cells were incubated with 10 µg/ml BODIPY-QS-21 for 1 h at either 4°C or 37°C, and fluorescence was visualized by vital confocal microscopy. **(B)** *Surface stripping*: THP-1 cells (*n* = 3) were incubated at 4°C for 1 h with 10 µg/ml ^14^C-QS-21, then extensively washed (Ctrl) or further stripped at 4°C by trypsin or heparin sulfate. **(C)** *Endocytosis*: Control or ATP-depleted THP-1 (*n* = 3) cells were incubated at 37°C for 4 h with 10 µg/ml ^14^C-QS-21, then stripped by trypsin as at **(B)**. Parallel cells were incubated at 4°C. Statistical significance was determined by a one-way ANOVA followed by Tukey’s multiple comparisons test. **(D)** *Cholesterol dependence*: THP-1 cells (*n* = 2–3) were cholesterol depleted or not by treatment with 2.5 mM methyl-β-cyclodextrin (MβCD) for 4 h, extensively washed, then incubated with ^14^C-QS-21 for 1 h or ^125^I-transferrin or ^125^I-cholera toxin/B subunit (CTxB) for 15 min, surface-stripped by trypsin and counted. Statistical significance was determined by a two-way ANOVA followed by Sidak’s multiple comparisons test. **(E,F)** moDCs (*n* = 8) were treated with the indicated concentration of MβCD for 1 h, extensively washed and stimulated with QS-21 or MPL for 4 h. IL-6 **(E)** and TNF **(F)** mRNA abundance was measured by qPCR and normalized to β-actin expression. Statistical significance was determined by a Wilcoxon matched-pairs signed-rank test. The asterisks depict significant differences between 5–10 mM MβCD and 0 mM MβCD for QS-21 stimulated cells.

Next, we analyzed the mode of capture and subcellular localization of QS-21. In order to have a sufficient number of cells, phorbol 12-myristate 13-acetate (PMA)-differentiated monocytic THP-1 cells were used for both biochemical and fractionation experiments. Cells were first incubated at 4°C with the fluorescent analog BODIPY-QS-21 (formulated into cholesterol-containing liposomes), which arrests endocytosis and limits passive diffusion. At this temperature, the fluorescence signal was exclusively detected at the cell surface. In contrast, after incubation at 37°C, QS-21 became detectable in distinct cytoplasmic, mostly perinuclear puncta (Figure 2A). To separately measure surface binding vs. intracellular uptake, we validated a surface stripping assay after incubation with ^14^C-QS-21 (also formulated into cholesterol-containing liposomes) at 4°C. Surface digestion with trypsin or treatment with heparin sulfate virtually abolished cellular uptake, indicating that surface binding depended on plasma membrane proteins and electrostatic interactions (Figure 2B). To further exclude passive diffusion as mechanism of intracellular uptake, the ATP content was depleted by preincubation with azide/deoxyglucose and intracellular (trypsin-resistant) uptake of ^14^C-QS-21 was measured at 37°C. Similarly to incubation at 4°C, energy depletion abolished the intracellular uptake of QS-21 at 37°C, demonstrating an active process requiring cellular energy and resulting in its accumulation in endocytic structures (Figure 2C).

Given that saponins interact with cholesterol (8), we next examined the role of membrane cholesterol in the endocytosis of ^14^C-QS-21. Minimal depletion of membrane cholesterol (<10% loss) by the addition of 2.5 mM (low-dose) methyl-β-cyclodextrin (MβCD), followed by extensive washing to eliminate residual MβCD, inhibited ^14^C-QS-21 uptake by ~50%, contrasting with the absence of effect on the intracellular uptake of either ^125^I-transferrin or ^125^I-cholera toxin B markers for clathrin- and caveolae/lipid-raft-mediated endocytosis (Figure 2D). Given that QS-21 endocytosis involves cholesterol, the effect of membrane cholesterol extraction on the response of moDCs to QS-21 was then investigated. Pretreatment of moDCs with MβCD inhibited their response to QS-21 but not to MPL, indicating that membrane cholesterol was a QS-21-specific requirement (Figures 2E,F). These results show that QS-21 was actively endocytosed in a cholesterol-dependent manner and that membrane cholesterol was crucial for the response to QS-21.

### QS-21 Mediates Syk Kinase Activation That Is Essential for Response of moDCs

**Figure 3:**
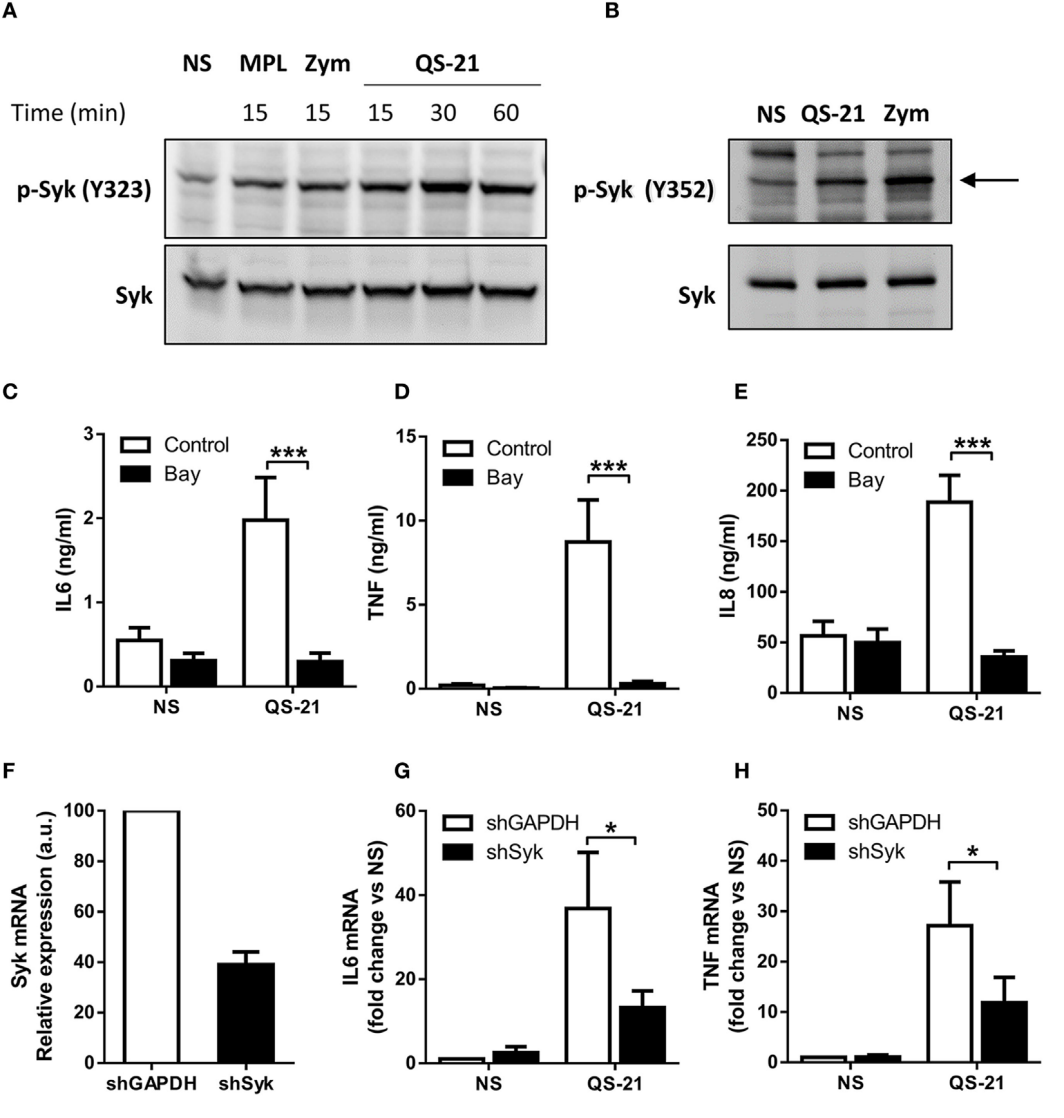

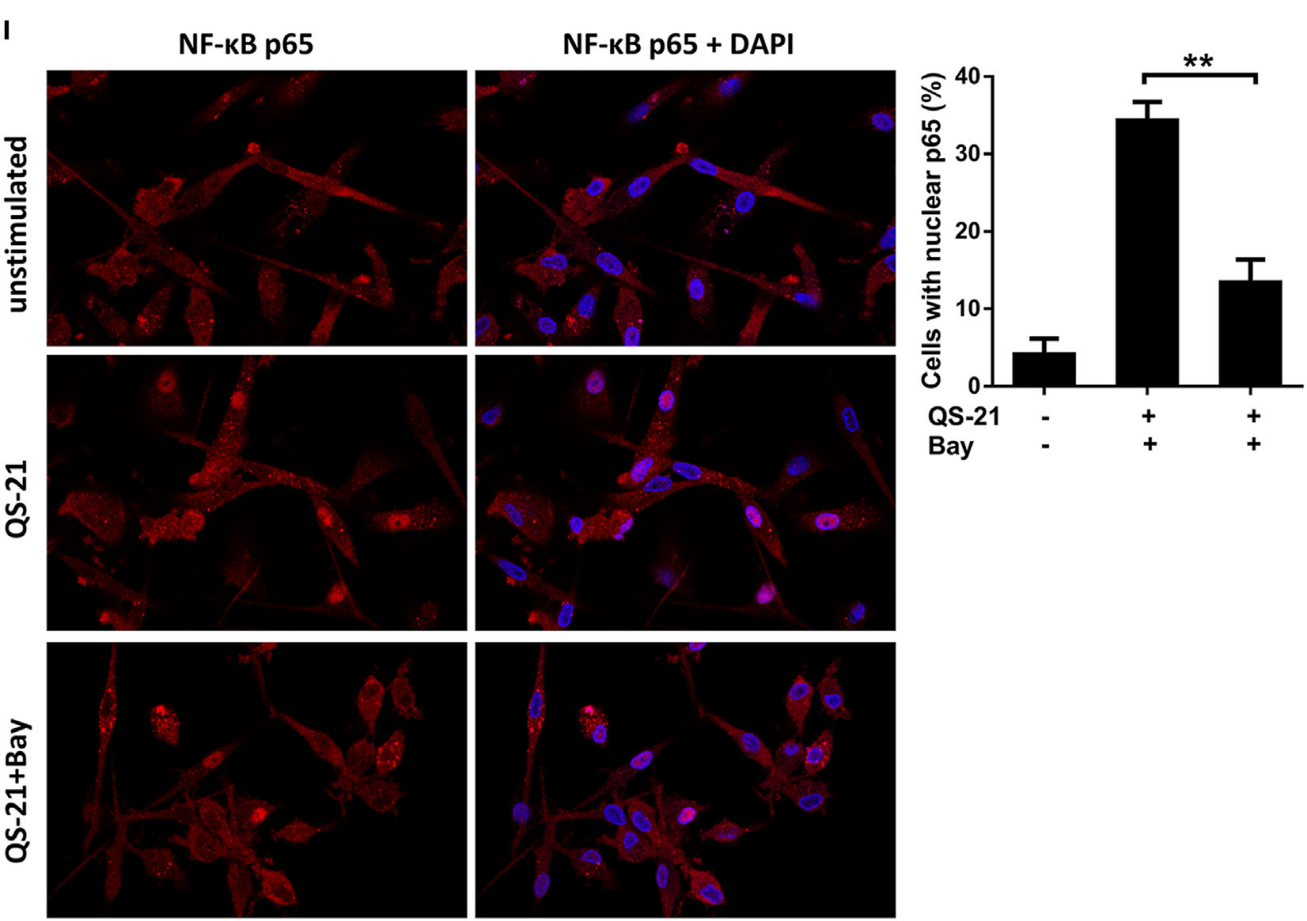
**Syk activation and signaling is indispensable for response to QS-21 in moDCs. (A,B)** Immunoblot analysis of cytoplasmic extracts of moDCs stimulated with QS-21, MPL, or Zymosan (Zym) for the indicated durations. Blots were probed sequentially with pY323-SYK **(A)** or pY352-SYK **(B)**, followed by total Syk. **(C–E)** moDCs were treated with Bay 61-3606 (10 µM) for 1 h and stimulated with QS-21 (10 µg/ml) for 24 h. IL-6 **(C)**, TNF **(D)**, and IL-8 **(E)** were quantified in the supernatant by ELISA. Statistical significance was determined by a Wilcoxon matched-pairs signed-rank test. **(F–H)** moDCs were transduced with lentiviruses coding for shRNAs targeting GAPDH or Syk, and Syk **(F)**, IL-6 **(G)**, and TNF **(H)** mRNA expression were measured by qPCR and normalized against β-actin expression. Statistical significance was determined by a Wilcoxon matched-pairs signed-rank test. **(I)** moDCs were seeded on coverslips, treated with Bay 61-3606 (10 µM) for 1 h and stimulated with 10 µg/ml QS-21 for 2 h. NF-κB p65 was visualized by immunofluorescence and nuclear p65 was quantified using ImageJ software. Data are representative of three experiments. Statistical significance was determined with a non-parametric Mann–Whitney *t*-test.

Membrane alterations, and more specifically membrane cholesterol remodeling, can induce activation of the Syk kinase independently of receptor ligation (32). We therefore investigated the role of this kinase in the response to QS-21. Dendritic cells were activated and Syk phosphorylation was measured by Western blotting. When cells were stimulated with QS-21, Syk phosphorylation increased on tyrosine 323 and 352, two known sites of Syk autophosphorylation (33), to a comparable extent as in response to MPL or zymosan, a known inducer of Syk phosphorylation (Figures 3A,B). Blockade of Syk activity by BAY 61-3606 (34) inhibited response to QS-21 in moDCs, as seen by the reduction of TNF, IL-6, and IL-8 secretion (Figures 3C–E). Since pharmacological compounds can lack specificity, Syk expression was also inhibited by shRNA interference. CD14^+^ monocytes were transduced during their differentiation into moDCs with lentiviral particles encoding Syk or GAPDH shRNAs, as previously described (22). Syk mRNA knockdown, measured by quantitative PCR, was greater than 50% when compared to cells expressing an shRNA specific to GAPDH (Figure 3F). Syk knockdown strongly decreased both IL-6 and TNF mRNA expression induced by QS-21, confirming the results obtained with the inhibitor (Figures 3G,H). Finally, the effect of Syk inhibition on NF-κB activation was investigated by observing p65 nuclear translocation. Syk inhibition by BAY 61-3606 strongly decreased QS-21-induced p65 translocation to the nucleus, indicating that Syk activity is required for both NF-κB activation and cytokine production (Figure 3I).

### Lysosomal Destabilization Mediates the Response of Human DCs to QS-21

**Figure 4:**
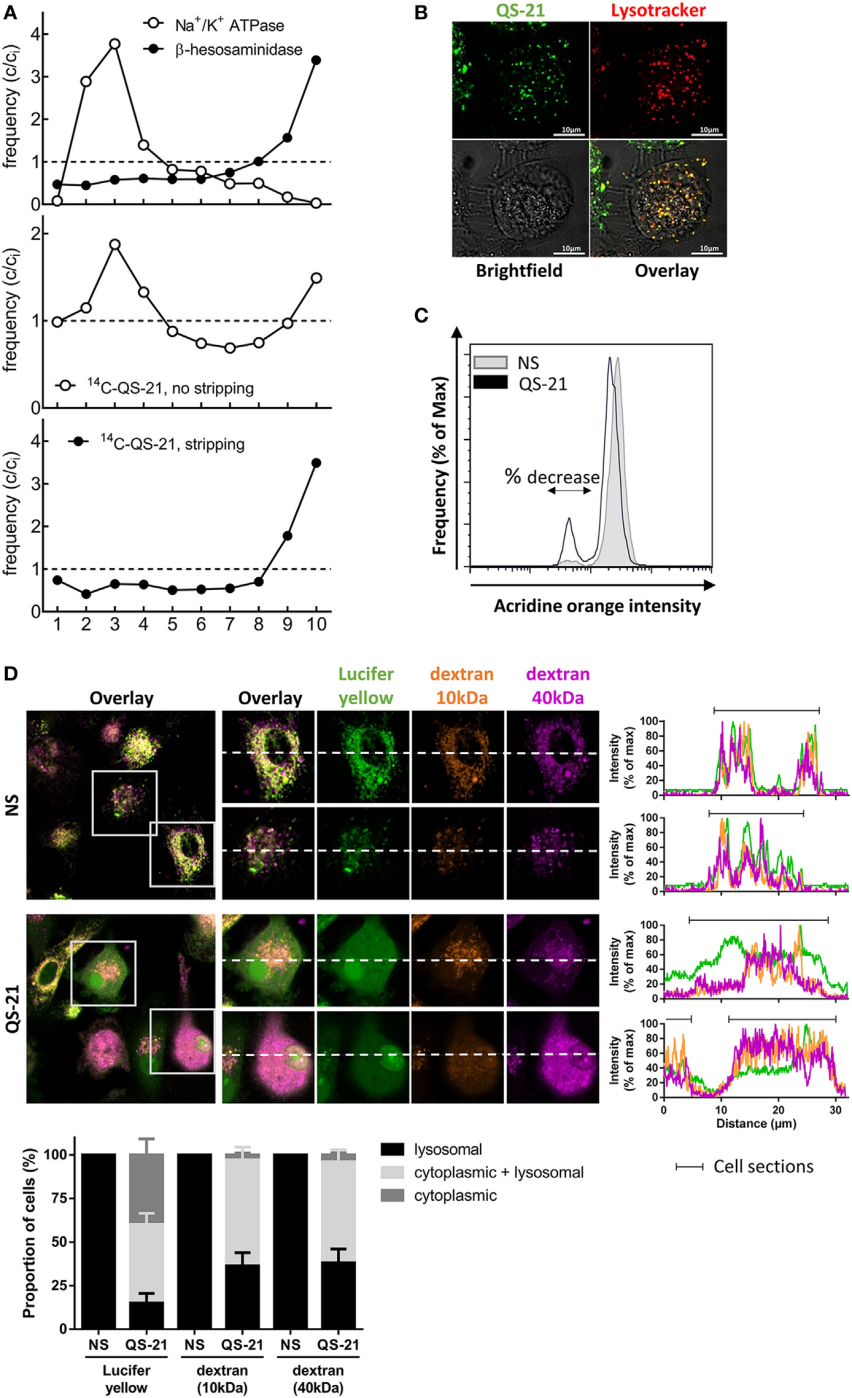
**QS-21 accumulates in lysosomes and promotes their destabilization**. **(A)** Differentiated THP-1 cells were pulsed with ^14^C-QS-21 for 4 h followed by an overnight chase, and postnuclear particles were resolved by density gradients fractionation. Na+/K+-ATPase identifies the plasma membrane and *N*-acetyl-β-hexosaminidase activity identifies lysosomes. The abscissa axis represents the different fractions. The ordinate reflects enrichment (*C*/*C_i_* > 1) or depletion (*C*/*C_i_* < 1) vs. initial abundance (*C_i_* = 1), indicated by dotted lines. **(B)** Differentiated THP-1 cells were co-incubated with BODIPY-QS-21 and Lysotracker Red then analyzed by vital confocal microscopy. **(C)** moDCs were incubated with 1 µg/ml acridine orange (AO), washed, and stimulated for 2 h with QS-21 (10 µg/ml). AO fluorescence was quantified by flow cytometry and a 610 nm filter. Cells with decreased AO fluorescence are indicated with the double arrow. One representative donor of 3 is shown. **(D)** moDCs were incubated with Lucifer Yellow, 10 kDa dextran-Alexa633 and 40 kDa dextran-Texas Red for 16 h then stimulated for QS-21 for 4 h. The localization of fluorescent markers was observed by confocal microscopy. The proportion of cells with punctate (lysosomal), diffuse (cytoplasmic) or both (punctate + diffuse) signal for the different fluorescent markers was determined with ImageJ. The data are representative of four donors.

The punctate pattern of cells incubated with BODIPY-QS-21 (Figure 2A) suggested that QS-21 may be confined to a specific subcellular compartment or structure. We therefore established the subcellular distribution of ^14^C-QS-21 by density gradient fractionation in THP-1 cells after 4 h of pulse and overnight chase. Equilibration of postnuclear particles in Percoll gradients fully resolved the plasma membrane (Western blotting for Na^+^/K^+^-ATPase, buoyant fractions) from lysosomes (*N*-acetyl-β-hexosaminidase activity, dense fractions) (Figure 4A). In the absence of surface stripping by trypsin, the density distribution of QS-21 was bimodal between these two positions in the gradient, indicating two pools at the plasma membrane and lysosomes, respectively. When cell surface proteins and associated material were stripped by trypsin digestion, the intracellular pool of QS-21 perfectly co-distributed with dense lysosomes. Colocalization of BODIPY-QS-21 with LysoTracker Red confirmed that internalized QS-21 was concentrated in lysosomes (Figure 4B).

**Figure 5:**
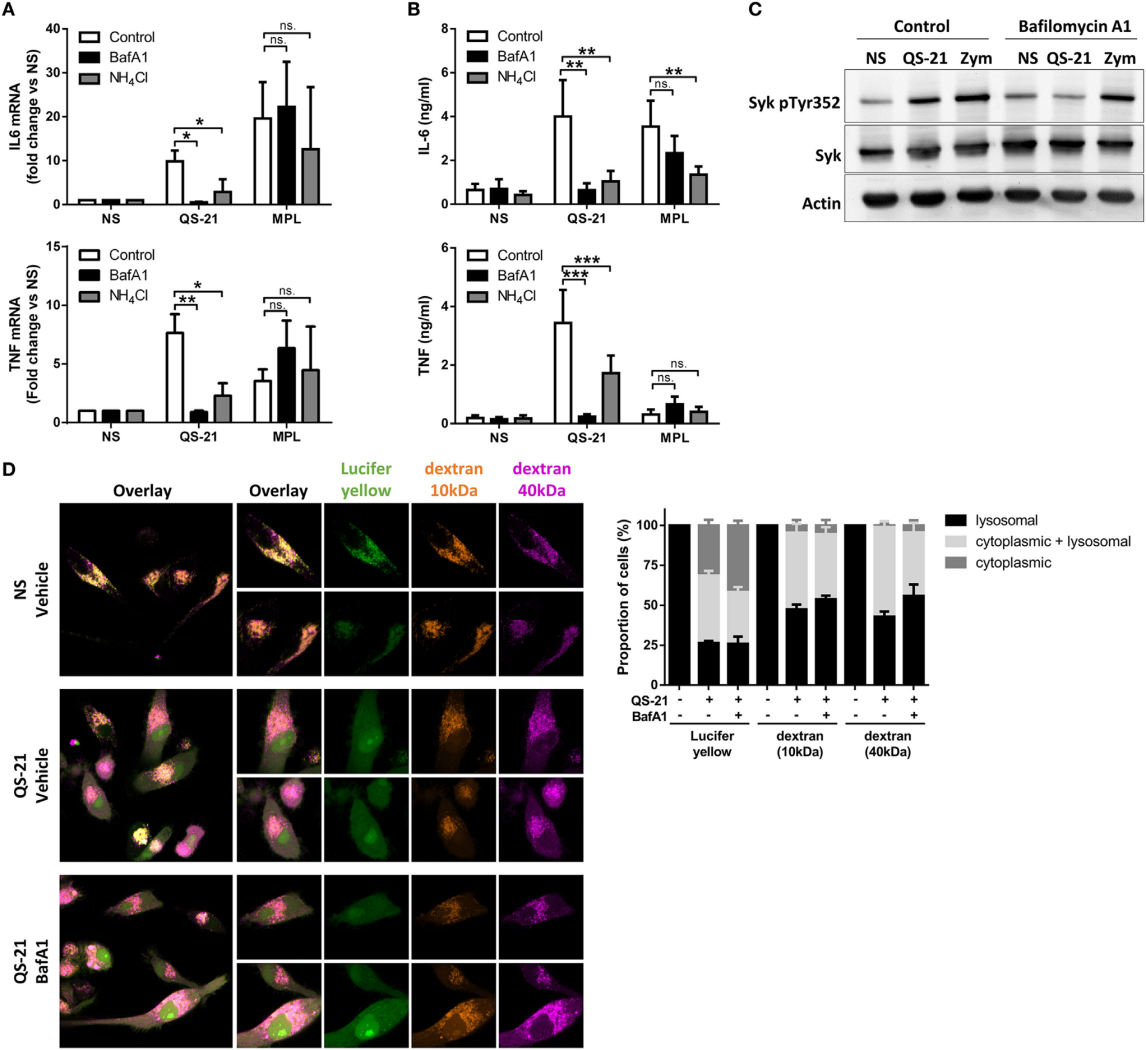
**QS-21-mediated dendritic cell activation and Syk phosphorylation depend on lysosomal maturation**. **(A,B)** moDCs were treated with bafilomycin A1 (BafA1—250 nM) or NH_4_Cl (10 mM) for 1 h and stimulated with QS-21 (10 µg/ml) or MPL (1 µg/ml) for 4 h **(A)** or 24 h **(B)**. TNF and IL-6 mRNA (A) and protein (B) were quantified by qPCR (*n* = 8) and ELISA (*n* = 11), respectively. Statistical significance was determined by two-way ANOVA followed by Tukey’s multiple comparisons test. **(C)** moDCs were treated with BafA1 for 1 h and stimulated with QS-21 (10 µg/ml) or zymosan as a positive control (Zym—50 µg/ml) for 2 h. Phospho-Syk (Y352), Syk, and β-actin were detected by sequential western blotting. One representative donor of 2 is shown. **(D)** moDCs were incubated with Lucifer Yellow, 10 kDa dextran-Alexa633, and 40 kDa dextran-Texas Red for 16 h then pretreated with BafA1 or DMSO (vehicle) and stimulated with QS-21 for 4 h. The localization of fluorescent markers was observed by confocal microscopy. The proportion of cells with punctate (lysosomal), diffuse (cytoplasmic), or both (punctate + diffuse) signals for the different fluorescent markers was determined with ImageJ. The data are representative of two donors.

Since saponins can interact with cell membranes and induce pore formation (8), we next examined the effect of QS-21 on lysosomal integrity. AO is a lysosomotropic dye that emits red fluorescence when concentrated under acidic conditions. moDCs preincubated with AO showed a rapid, yet partial decrease in red fluorescence upon stimulation with QS-21, indicating loss of lysosomal acidification or membrane integrity (Figure 4C). To investigate whether this second possibility could be due to saponin-mediated pore formation, moDCs were incubated with fluorescent endocytic markers of different molecular weights. In untreated cells, Lucifer Yellow, a very small yet membrane-impermeant lysosomal endocytic tracer (LY; 522 Da), as well as two dextrans of different sizes (10 kDa Alexa647-Dextran and 40 kDa Texas Red-Dextran) co-localized in lysosomes after 16 h. After stimulation with QS-21, tracers were detected in the cytosol (80% of cells for the small LY and 60% for the two dextrans) (Figure 4D). Furthermore, in 40% of cells, LY fluorescence showed only a diffuse signal with loss of puncta, while both dextrans were still visible in lysosomes. QS-21 therefore partially permeabilized lysosomal membranes with a selectivity in pore size, resulting in widespread cytosolic diffusion of the small tracer but only partial diffusion of the two dextran polymers. To determine whether low lysosomal pH was a crucial factor for the response to QS-21, lysosomal acidification was blocked by inhibition of the vacuolar ATPase by bafilomycin A1 (BafA1), or by incubation with the weak base ammonium chloride (NH_4_Cl). Incubation of moDCs with either agents lessened both QS-21-induced IL-6 and TNF mRNA transcription (Figure 5A) and cytokine release (Figure 5B). No such effect was observed upon stimulation with MPL, excluding a defect in IL-6 secretion. BafA1 treatment also inhibited Syk Y352 phosphorylation induced by QS-21 but not by zymosan, strongly suggesting that QS-21 induced Syk phosphorylation takes place downstream of lysosomal destabilization (Figure 5C). Finally, pretreatment of cells with BafA1 had no effect on QS-21-induced relocation of either LY or dextrans from the lysosomes to the cytosol, indicating that lysosome pore formation occurred even if acidification was inhibited (Figure 5D). These results suggest that acidic pH in lysosomes and lysosomal membrane permeabilization by QS-21 are necessary for the activation of moDCs.

### Lysosomal Cathepsin B Mediates the Response to QS-21 in Human Dendritic Cells

**Figure 6:**
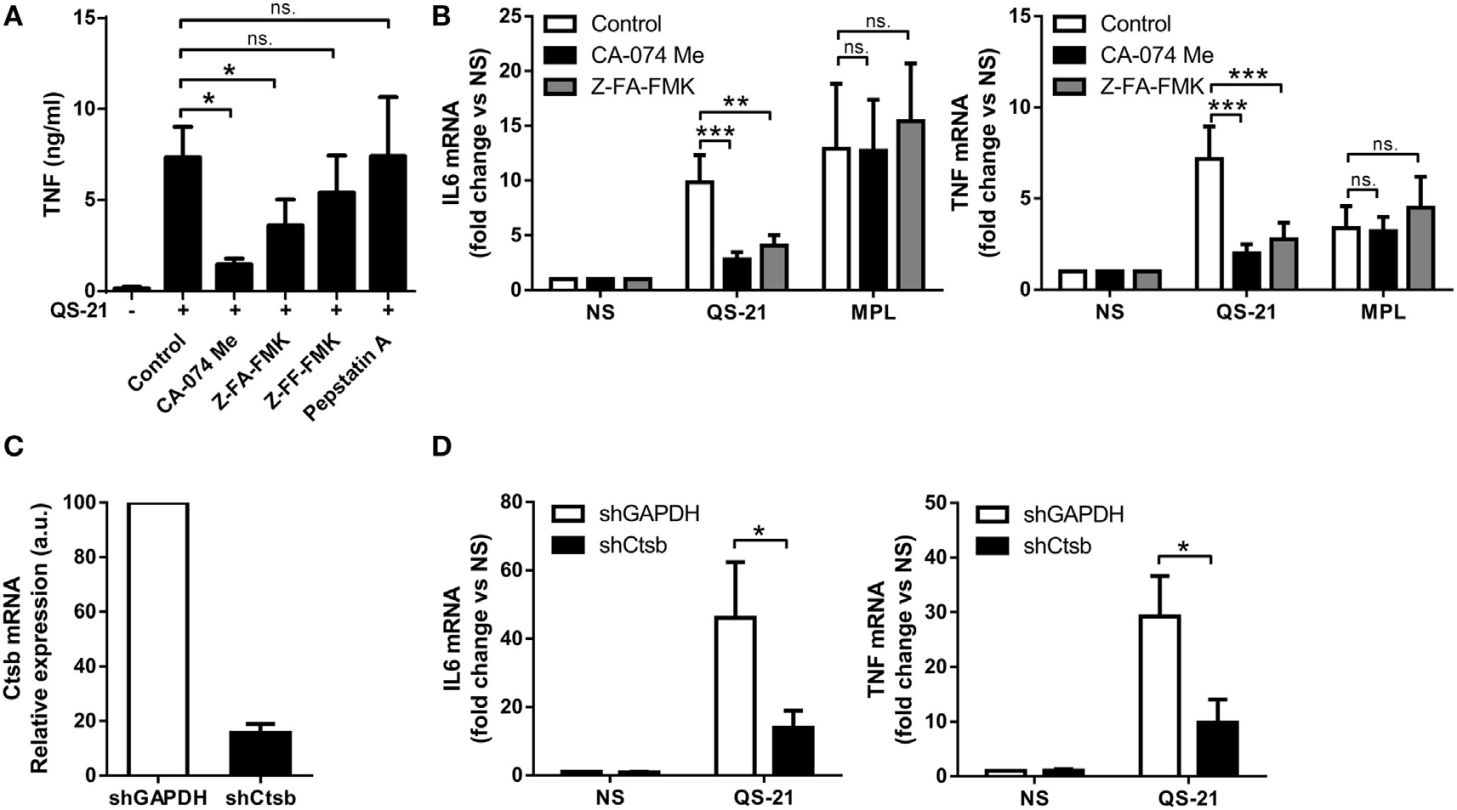
**Cathepsin B activity is required for an optimal response to QS-21**. **(A)** moDCs were incubated with CA-074 Me (cathepsin B inhibitor), Z-FA-FMK (cathepsin B inhibitor), Z-FF-FMK (cathepsin B/L inhibitor), or pepstatin A (cathepsin D, cathepsin E, and pepsin inhibitor) for 1 h and stimulated with QS-21 for 24 h. TNF release into the supernatant was measured by ELISA. Statistical significance was determined with a Friedman test followed by Dunn’s multiple comparison test. **(B)** moDCs were pretreated with CA-074 Me or Z-FA-FMK for 1 h and stimulated with QS-21 or MPL for 6 h. IL-6 and TNF mRNA levels were quantified by qPCR and normalized to β-actin mRNA levels. Statistical significance was determined by two-way ANOVA followed by Tukey’s multiple comparisons test. **(C,D)** moDCs were transduced with lentiviruses coding for shRNAs targeting GAPDH (shGAPDH) or cathepsin B (shCtsb), and cathepsin B **(C)**, IL-6 and TNF **(D)** mRNA expression were measured by qPCR and normalized against β-actin expression. Statistical significance was determined by a Wilcoxon matched-pairs signed-rank test.

Some lysosomal cathepsins are reported to activate NF-κB-mediated transcriptional events when released into the cytosol (35, 36). To screen for involvement of member(s) of this protease family in QS-21-mediated activation of moDCs, cells were pretreated with the following inhibitors before challenge with QS-21: CA-074 Me and Z-FA-FMK (cathepsin B); Z-FF-FMK (cathepsin L), or pepstatin A (cathepsin D, cathepsin E). Only the two first inhibitors significantly decreased QS-21-mediated TNF production, pointing to a specific involvement of cathepsin B in this response (Figure 6A). Cathepsin B inhibition with either CA-074 Me or Z-FA-FMK also inhibited QS-21-induced TNF and IL-6 mRNA expression with no effect on MPL-induced gene expression, indicating that the effect was transcriptional and specific to QS-21 (Figure 6B). Finally, shRNA-mediated knockdown of cathepsin B strongly decreased the expression of both TNF and IL-6 mRNAs, confirming that cathepsin B expression is essential for QS-21-mediated cytokine production in human DCs (Figures 6C,D).

### Cathepsin B Is Essential for Optimal Antigen-Specific QS-21-Mediated T Cell Responses *In Vivo*

**Figure 7:**
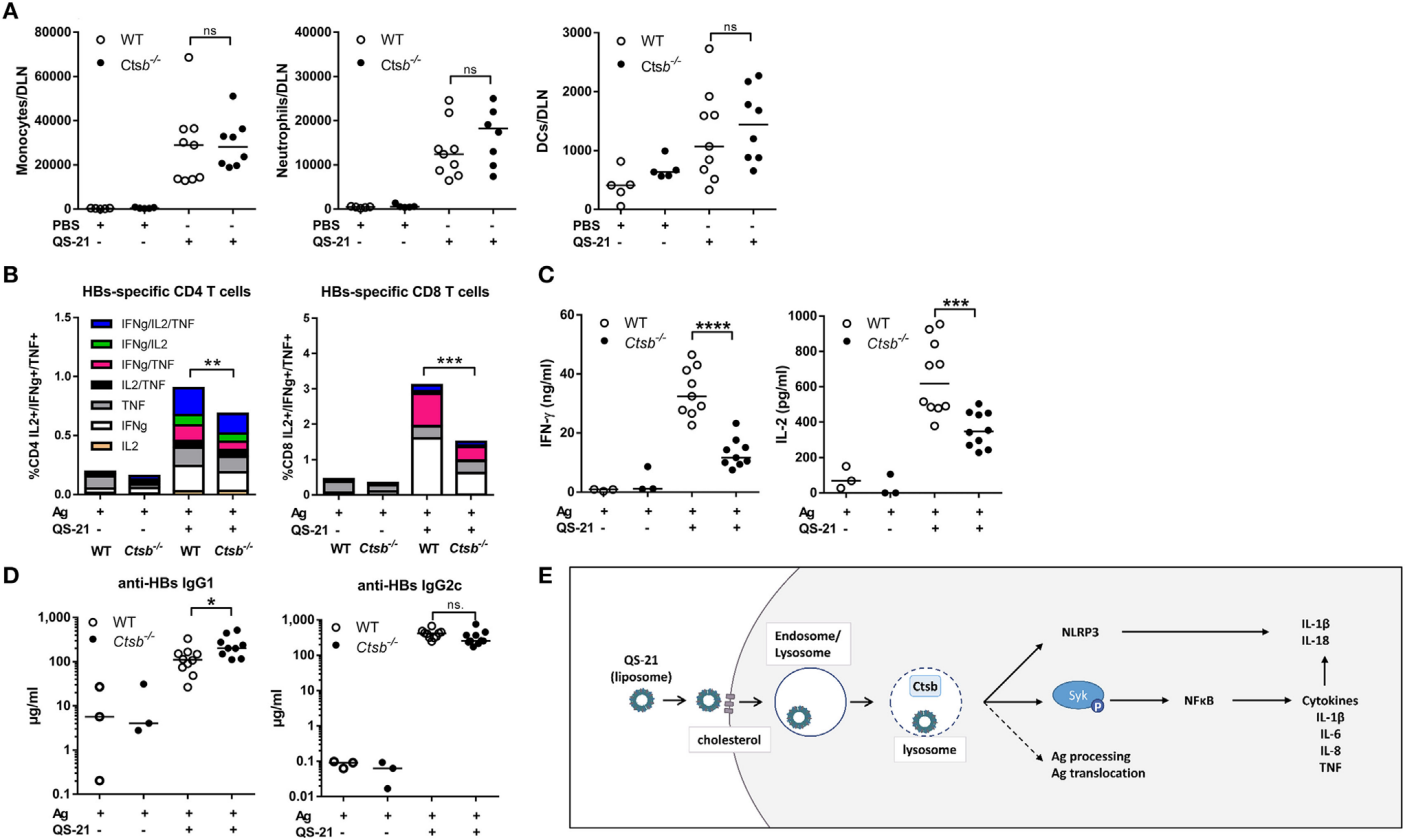
**QS-21 elicited antigen-specific CD4 and CD8 T cell responses are dependent on cathepsin B**. **(A)** Monocyte (CD11b^+^ Ly6C^+^ Ly6G^−^), neutrophil (CD11b^+^ Ly6C^int^ Ly6G^+^), and dendritic cell (CD11c^hi^ MHCII^hi^) recruitment to the draining lymph node 24 h post intramuscular immunization with PBS or QS-21 assessed by flow cytometry. **(B)** WT and cathepsin B (Ctsb)-invalidated mice were immunized at day 0 and day 14 with hepatitis B surface (HBs) antigens or with HBs antigens adjuvanted with QS-21. At day 21, median cytokine production of HBsAg-specific splenic CD4 and CD8 T cells were evaluated by intracellular staining following *ex vivo* restimulation with antigenic peptides (Ag: *n* = 6, QS-21: *n* = 20). **(C)** IFN-γ and IL-2 production by HBs-specific spleen cells were measured by ELISA following restimulation with HBs peptides (Ag: *n* = 3, QS-21: *n* = 10). **(D)** Anti-HBs IgG1 and IgG2c titers in the serum at day 21 were measured by ELISA (Ag: *n* = 6, QS-21: *n* = 26). Each dot represents one mouse, and the horizontal bar represents the geometric mean. Statistical significance was determined by a non-parametric Mann–Whitney *t*-test. The data represent the pooled results of two independent experiments. **(E)** Proposed model: QS-21 is endocytosis via a cholesterol-dependent mechanism. It then traffics to lysosomes and induces their destabilization. This can induce inflammasome activation and Syk- and cathepsin B-dependent cell activation and cytokine production in moDCs. Lysosomal destabilization could also affect antigen processing and antigen translocation to the cytosol.

To assess the role of cathepsin B in the adjuvant effect of QS-21 *in vivo*, we first evaluated the recruitment of inflammatory cells to the lymph node draining the site of QS-21 injection. In both WT and cathepsin B KO mice, QS-21 induced the recruitment of monocytes and neutrophils, and to a lesser extent dendritic cells 24 h post-immunization (Figure 7A), suggesting that other pathways may contribute to these early events *in vivo*. Next, to evaluate a possible role of cathepsin B in adaptive responses, WT or cathepsin B KO mice were immunized intramuscularly with either HBsAg or HBsAg adjuvanted with QS-21. HBsAg is the antigen used in the Engerix-B^TM^ vaccine. Mice were immunized following a prime (at day 0)-boost (at day 14) regimen and the Ag-specific adaptive responses were measured at day 21. The frequency of cytokine (IL-2, TNF, and IFN-γ)-producing Ag-specific T cells, measured by flow cytometry following *ex vivo* restimulation with antigenic peptides, was significantly lower in cathepsin B-deficient mice than in their wild-type counterparts for both HBsAg-specific CD4 and CD8 T cells (Figure 7B). A strong decrease in IFN-γ and IL-2 secretion was also observed in the supernatants of cathepsin B-deficient splenocytes in the same experimental setting (Figure 7C). However, despite these marked defects in both CD4 and CD8 Ag-specific T cells responses, antibody titers were not significantly reduced (Figure 7D).

## Discussion

QuilA saponins, and QS-21 in particular, are well-known adjuvants but, excluding NLRP3 inflammasome activation, little is known about the molecular and cellular mechanisms leading to their adjuvant effect. Here, we demonstrate that cholesterol-dependent endocytosis is required to induce human moDC activation and that QS-21 accumulates in lysosomes and causes lysosomal membrane permeabilization. Cell activation depends on the activity of the Syk kinase and of cathepsin B (Figure 7E). Finally, we established that cathepsin B participates in the adjuvant properties of QS-21 on both CD4 and CD8 antigen-specific-T cell responses *in vivo*.

QS-21 is a saponin with specific affinity for cell membrane cholesterol that can induce pore formation therein (8). Co-formulation with cholesterol is required to avoid toxicity when used as injectable (10). In this study, we used QS-21 formulated in liposomes containing cholesterol and thereby devoid of any measurable lytic activity. Membrane cholesterol in the target cells was required for both cell entry and activation, suggesting that either QS-21 is transferred from liposomal cholesterol to the cell membrane-associated cholesterol or that the whole liposome containing QS-21 is endocytosed via a cholesterol-dependent mechanism. Partial depletion of membrane cholesterol did not impact the endocytosis of either transferrin (TfR) or cholera toxin B (CTxB), arguing against clathrin-dependent and caveolin/lipid raft-dependent mechanisms for endocytosis of liposomal QS-21 (37). Cell entry could instead be mediated through cholesterol-dependent macropinocytosis as shown for several viruses (38–42). Macropinocytosis of viruses can be receptor mediated (42), which could explain the surface protein dependence we observed for QS-21. Irrespective of the mechanism of entry, QS-21 was eventually transferred to and concentrated in dense lysosomes where it promoted lysosomal destabilization and the potential release of lysosomal content. Because particles of up to 40 kDa could leak out of lysosomes upon incubation with QS-21, it is likely that pores were formed in the lysosomal membrane. Several mechanisms could explain why QS-21 could form pores in lysosomal membranes but not in plasma membranes. On the one hand, due to the lysosomal accumulation and lengthy retention of QS-21, cholesterol may be preferentially extracted by QS-21 in these organelles. Reduced lysosomal membrane cholesterol has been linked to increased permeability to positive ions leading to osmotic imbalance and lysosomal destabilization (43–45). Lysosome destabilization by listeriolysin and lysosomotropic detergents is dependent on acid-driven conformational changes occurring in this organelle (46, 47). This does not seem to be the mechanism for QS-21, as inhibition of lysosome acidification did not affect QS-21-mediated pore formation. Interestingly, inhibition of lysosome acidification also inhibits the response to QS-21 in human PBMCs. Indeed, treatment with chloroquine inhibited QS-21-induced production of IL-6 by PBMCs, although no effect of chloroquine administration was observed on T cell or antibody responses in healthy adults immunized with AS01-adjuvanted antigens (48).

QS-21-mediated pore formation resulted in the translocation of macromolecules from lysosomes to the cytosol and induced activation marker upregulation and cytokine transcription. This complex activation pathway is most likely the reason for the different expression kinetics observed by PCR for cytokines induced by QS-21 when compared to MPL. Furthermore, the genes upregulated specifically by QS-21 identified in the microarray included *EGR1, RGS1, NR4A2*, and *DUSP1* that are known TLR4-induced primary response genes. These genes are expressed as rapidly as 15 min following TLR4 stimulation and their expression returns to baseline after 2–3 h (25–28). It is therefore likely that after 4 h, the time point chosen for the microarray analysis, the expression of these genes, had returned to baseline after stimulation with MPL but not yet with QS-21.

Other groups have reported that lysosomal permeabilization by compounds including Alum and QuilA activates the inflammasome, although pro-IL-1β expression required priming with a TLR-ligand (15, 49, 50). In contrast, here we show that QS-21 directly promoted the expression of pro-IL-1β and other cytokines. This discrepancy between our study and previous work may be due to the cell type studied or to the formulation. Indeed, a recent study has shown that stimulation with unformulated (i.e., not formulated with liposomes) QS-21 does not lead to the direct activation of murine bone marrow-derived dendritic cells or macrophages (14). It is possible that formulation of QS-21 with cholesterol-containing liposomes allows for better lysosomal targeting and activation of alternative signaling pathways. Cell type specificity has also been observed for saponin-dependent translocation of endocytosed antigens, as it occurs in human moDCs but not in human monocytes or macrophages (6). Human dendritic cells derived from monocytes *in vitro* have an increased capacity for lysosomal proteolysis when compared to other human or mouse dendritic cells and express high levels of lysosomal cathepsins B, D, L, and S (51). This distinctive characteristic may explain why saponins could promote both antigen translocation in and direct activation of human moDCs.

We have indeed shown that cathepsin B expression and activity are critical for the response of moDCs to QS-21. Furthermore, lysosomal cysteine proteases, such as cathepsins B and L, are involved in inflammasome activation downstream of lysosomal destabilization following stimulation with saponins (14, 15). The exact mechanism by which cathepsin B could promote the transcription of pro-inflammatory cytokines remains elusive, although a role for cathepsins in NF-κB activation has already been described. The synthetic double-stranded RNA, poly IC, can induce lysosomal destabilization and cathepsin D release into the cytosol, where it mediates the cleavage of caspase-8, which is important for increased NF-κB activity (36). In our experiments however, cathepsin B did not seem to cleave caspase-8, as no cleavage fragments were detectable in cells stimulated with QS-21 (data not shown). Cathepsin B has nevertheless multiple other potential lysosomal and cytoplasmic targets.

*In vivo*, cathepsin B was not involved in Ag-specific antibody responses promoted by QS-21 or innate cell recruitment to the draining lymph node, yet cathepsin B-deficient mice showed decreased HBsAg-specific CD4 and CD8 T cell responses. This discrepancy could be due to a general defect in antigen presentation. Indeed, cathepsin B activity has been linked to antigen processing, which could affect antigen presentation on both class I and class II major histocompatibility complexes (52–56). Interestingly, ISCOMATRIX was shown to induce antigen translocation from lysosomes to the cytosol, thereby facilitating proteasome-independent cross-priming (6). We have also shown that QS-21 promotes pore formation in lysosomal membranes allowing the release of macromolecules of up to 40 kDa into the cytosol. Cathepsin B could partially degrade protein antigens in the lysosome and the fragments could be released into the cytosol by QS-21 for cross-presentation. It is, however, unlikely that this mechanism contributes to lessened QS-21-elicited Ag-specific CD8 T cell responses observed in cathepsin B-deficient mice, since QS-21 and antigens display different localizations and pharmacokinetic properties after immunization (16).

We have also identified Syk as a key signaling molecule for the response of moDCs to QS-21. Indeed, Syk was phosphorylated following QS-21 stimulation and Syk knockdown or pharmacological inhibition blocked NF-κB activation and cytokine production. Syk is a tyrosine kinase generally associated with ITAM-motif containing receptors (57), and can also play a role in lysosomal function in B cells. For example, B cell receptor cross-linking causes Syk-dependent changes in lysosomal pH, which lead to apoptosis (58). However, in our system, Syk was found to act downstream of lysosomal function, as bafilomycin A1 blocked QS-21-mediated Syk phosphorylation. Syk may therefore play a role of lysosomal integrity sensor and auto-phosphorylate following lysosomal membrane damage, a possibility that has already been suggested (59). Since Syk kinase can be activated by plasma membrane lipid alterations, it is possible that a similar mechanism could occur at the lysosomal membrane (32, 60). However, neither Syk relocation to nor phosphorylation at the lysosomal membrane could be detected by confocal microscopy in our system (data not shown). Nevertheless, Syk could promote cathepsin B proteolytic activity as has been shown in other models (61). In summary, we describe a novel lysosome-dependent pathway (Figure 7G) that contributes to the immunostimulatory properties of a clinically approved saponin-based vaccine adjuvant. This knowledge may help the rational development of adjuvants which could be instrumental to the success of future vaccination strategies.

## Ethics Statement

Ethics committee (Erasme hospital) (comité d’éthique de la faculté de médecine, 808 route de Lennik, B-1070 Brussels, Belgium). Animal studies were approved by the local animal welfare committee (commission d’éthique en expérimentation animale du Biopôle ULB-Charleroi). Buffy coats were obtained from local blood donations by the Red Cross.

## Author Contributions

IW conducted most of the experiments. SD, FN, and SW contributed to some experiments. ST provided technical help for the experiments. IW, SD, VB, SW, and PC analyzed the data. RG and AE provided input for research design and interpretation. TR bred and provided *Ctsb^−/−^* mice. IW, SG, PC, and AMD designed the study and wrote the manuscript. All the authors were involved in critically revising the manuscript for important intellectual content. All the authors had full access to the data and approved the manuscript before it was submitted by the corresponding author.

## Conflict of Interest Statement

All authors have declared the following potential interests. SW, VB, RG, AE, and AMD are, or were at the time of the study, employees of the GSK group of companies. SW, VB, AE, and AMD own GSK stocks. The other authors report no financial conflict of interest.
